# Supplementary material for: Explaining public dental service utilization: A theoretical model
Source: PLoS One. 2023 Sep 1;18(9):e0290992. doi: 10.1371/journal.pone.0290992 (PMC10473501; doi:10.1371/journal.pone.0290992)
Supplement: Attachment — Submitted filename: cover letter.docx [file pone.0290992.s001.docx]

Dear Editor Isabel Cristina Gonçalves Leite,

We would like to express our gratitude for the reviewers and the academic editor's thorough review of our manuscript " Explaining public dental service utilization: a theoretical model”. Their valuable insights and constructive feedback have significantly contributed to enhancing the quality and clarity of our work. We appreciate the time and effort invested in evaluating our submission. We are pleased to confirm that all the points raised by the academic editor and reviewers have been duly addressed and incorporated into the revised version of our manuscript.

1-Conceptualization of the Andersen Model:

We acknowledge the suggestion made by Reviewer 1 to include a conceptualization of the Andersen model in the introduction. We have revised the introduction to provide an overview of the Andersen Model. This addition enhances the context and helps readers better understand the theoretical framework of the theme.

2-Objective Refinement:

We thank Reviewer 2 for the insightful comments regarding refining our research objectives. In response to their suggestions, we have rephrased the objectives. These revised objectives enhance the precision of our research goals, contributing to a more focused and coherent presentation.

3-Reference List Review:

Furthermore, we would like to highlight that we have thoroughly reviewed our reference list to ensure its completeness and accuracy. Alterations have been made as necessary to ensure the citations' accuracy.

We wish to express our sincere appreciation to the academic editor and reviewers for their positive remarks and helpful guidance. Their expertise and constructive critique have significantly improved the quality and rigor of our manuscript.

Once again, we express our gratitude for the opportunity to revise and resubmit our manuscript. We are confident that the changes made in response to the feedback have strengthened our paper and aligned it more closely with the standards of PLOS ONE Journal.

Thank you for your consideration of our revised manuscript. We look forward to the possibility of its publication in PLOS ONE.

Sincerely,

Maria Helena Galvão, Ph.D

Professor at the Federal University of Pernambuco, Brazil.

Angelo Giuseppe Roncalli, Ph.D

Professor at the Federal University of Rio Grande do Norte, Brazil.
